# Alexithymic characteristics in pediatric patients with primary headache: a comparison between migraine and tension-type headache

**DOI:** 10.1186/s10194-015-0572-y

**Published:** 2015-11-25

**Authors:** M. Gatta, C. Spitaleri, U. Balottin, A. Spoto, L. Balottin, S. Mangano, P.A. Battistella

**Affiliations:** Department of Woman and Child Health, University of Padova, Padova, Italy; Department of Child and Adolescent Neuropsychiatry, University of Palermo, Palermo, Italy; Child Neuropsychiatry Unit, C. Mondino National Neurological Institute, Pavia, Italy; Child Neuropsychiatry Unit, Department of Brain and Behavioral Sciences, University of Pavia, Pavia, Italy; Department of General Psychology, University of Padova, Padova, Italy; Department of Philosophy, Sociology, Education, and Applied Psychology, Section of Applied Psychology, University of Padova, Padova, Italy

**Keywords:** Alexithymia, Toronto Alexithymia Scale, Alexithymia Questionnaire for Children, Tension-type headache, Migraine

## Abstract

**Background:**

Alexithymia is a personality construct characterized by difficulties in verbal emotional expression and a limited ability to use one’s imagination. Evidence of alexithymic characteristics was found in adults suffering from headache, while little is known about children. The aim of this study was to establish the prevalence of alexithymia in two different subgroups of children and adolescents suffering from primary headache. We also looked for correlation between alexithymia in children and in their mothers.

**Methods:**

This study involved 89 participants: 47 (11 males, 36 females, aged 8 to 17 years) suffering from tension-type headache (TTH), and 42 (18 males, 24 females, aged 8 to 17 years) suffering from migraine (M), based on the International Classification of Headache Disorders (ICHD 2013). A control group of 32 headache-free subjects (26 females and 6 males, aged 8 to17 years) was also considered. Two questionnaires were administered to measure alexithymia: the Alexithymia Questionnaire for Children to young patients and controls, and the Toronto Alexithymia Scale (TAS-20) to the mothers.

**Results:**

Higher rates of alexithymia emerged in the TTH group compared to the M group. In particular, TTH sufferers had difficulty identifying their feelings. The mothers of children with headaches didn’t score higher in alexithymia compared to other mothers. In the M and in the control group, there was a significant correlation between the rates of alexithymia in young people and in their mothers.

**Conclusions:**

To date no other study has investigated alexithymia in subgroups of primary headaches in developmental age. Our results suggest that patients suffering from TTH are more alexithymic than M patients. This pave the way to etiopathogenetic and clinical considerations, calling for a comprehensive and multidisciplinary approach to tackle the problem of headache.

## Background

The term alexithymia, which literally means “no words for feelings”, refers to a cognitive-affective impairment characterized by a limited capacity to experience and express emotions [1–4]. The most important features of alexithymia are: (a) a difficulty identifying feelings and distinguishing between feelings and the bodily sensations of emotional arousal; (b) difficulty describing feelings to others; (c) constricted imaginative processes; and (d) a stimulus-bound, externally oriented cognitive style [4]. Several theories of the etiology of alexithymia have been proposed. Some suggest that childhood events such as traumatic experiences and/or a dysfunctional parent–child relationship can contribute to alexithymia [5] while other works suggest relating nonverbal emotion recognition in alexithymia with a dysfunction of the right hemisphere [3, 6, 7]. Alexithymia can be linked to a deficiency in inter-hemispheric transfer of information from the right to the left hemisphere [8, 9], referred to by Hoppe as a “functional commissurotomy” [8]. Another theory attributes the pathogenesis of alexithymia to depleted dopamine levels in the anterior cingulate cortex or the orbitofrontal cortex [3, 10].

Several mechanisms may explain the way genetic factors contribute to individual differences in alexithymia: the genes potentially involved in the variability of alexithymic traits may influence the transcription of neuroreceptors or neurotransmitters, or may affect neural development [11]. Some family studies found a significant relation between mothers’ and fathers’ alexithymia scores and those of their children’s [12], suggesting a possible intergenerational transmission of alexithymia. However they cannot determine whether the clustering in the families observed is correlated to genetics or environmental influences. The design of a twin study is the most suited to disentangle the role of nature versus nurture, but to date there are few twin studies on alexithymia [12–15]. Two pioneering studies suggested important genetic influences in alexithymia [12, 13]. Nevertheless more recent studies have shown that genetic factors accounted for one third of the variance in TAS-20 scores, while unshared environmental factors accounted for most of the variation in alexithymia [14, 15].

The aim of this study is to investigate alexithymic characteristics of children and adolescents with primary headache and their mothers to verify whether there is a possible relationship between headache and emotional regulation, in particular alexithymia. A wide range of psychiatric disorders, in fact, seemed to be linked to primary headache and preexisting mental disorders are likely to increase the risk for the onset of headache [16]. Moreover, headaches are more common in families with a history of psychological disorders: mood and anxiety disorders run in families, just like headache symptoms, and they were shown to be more common in the parents of children with headache [17]. This points to the importance of assessing a broad range of psychiatric conditions and psychological traits, it includes alexithymia, in children and their parents, to discover important stressors and risk factors for the onset of headaches in genetically predisposed children [16–18].

There are few studies on the relationship between headache and alexithymia in adults, and still fewer on the issue related to children and adolescents [19]. Wise et al. [20] compared adult patients with migraine (M) or tension-type headache (TTH) with healthy controls, finding higher alexithymia scores in headache sufferers, irrespective of whether they had M or TTH. Yücel et al. [21] demonstrated higher levels of alexithymia in patients aged 18–65 suffering from episodic or chronic TTH than in healthy controls, once again with no difference in the severity of alexithymia between the cases experiencing episodic versus chronic TTH.

By means of a case–control study, we investigated a sample of pediatric patients suffering from primary headache (M or TTH), to detect any alexithymic characteristic. Assuming that the construct might depend on the relationship with the primary caregiver, the alexithymic dimension was also investigated in the patients’ mothers, comparing the levels of alexithymia obtained by the mothers with those of their children.

We therefore had three objectives:

- to establish whether a statistically significant difference in alexithymic traits exists among M patients, TTH patients and controls;
- to seek any statistically significant correlation between the alexithymic scores obtained by the children/adolescents and those obtained by their mothers;
- to identify any statistically significant association between age, gender and alexithymic score.

## Methods

### Participants

The study involved 47 TTH sufferers (11 males and 36 females) aged 8 to 17 years (mean 12.4 ± 2.3) and 42 M sufferers (18 males and 24 females) aged 8 to 17 years old (mean 13.1 ± 2,4), attending the Center for the Diagnosis and Treatment of Juvenile Headache at the Universities of Padova and Pavia. The inclusion criteria were TTH and M, diagnosed at least 6 months previously, with no pharmacological prophylaxis. The diagnosis was based on the ICHD (The International Classification of Headache Disorders 3^rd^ edition/beta version 2013) [22]. Within the TTH group, 20 (43 %) out of the 47 participants were diagnosed with chronic TTH, while 8 (17 %) and 19 (40 %) suffered from frequent episodic and infrequent episodic TTH respectively. Concerning the M group, 27 (64 %) out of the 42 patients were diagnosed with M without aura and 15 (36 %) with M with aura.

The control group (CG) consisted of 32 subjects (6 males and 26 females) aged 8 to 17 years (mean 11.8 ± 1.6) recruited among patients of three medical practitioners in Padova during a routine pediatric check-up at the practitioners’ outpatient offices. A semistructured clinical interview was conducted with the mother to collect information on clinical-medical history, including the family’s physiological, recent and remote, pathological medical history, focusing particularly on any symptoms of headache. A medical history of headaches or organic diseases and paternal or maternal familiarity for headaches were considered exclusion criteria to be included in the CG.

### Measures

Children answered an Italian form of the Alexithymia Questionnaire for Children (AQC) [23], a simplified version of the 20-Item Toronto Alexithymia Scale (TAS-20) [24]. The mothers were administered semistructured medical history interviews and had to answer the validated Italian version [25] of the Toronto Alexithymia Scale (TAS-20), the most widely used questionnaire for the assessment of alexithymia in adulthood. The TAS 20 is a 20-item self-rating questionnaire that measures the following factors: F1, difficulty identifying feelings; F2, difficulty describing feelings; and F3, externally oriented thinking. The results that emerged from the questionnaire were used to classify the subjects into three groups: non-alexithymic, borderline and alexithymic (score=/>61).

### Analisys

 The scores obtained on the AQC and on TAS-20 were analysed with ANOVA in order to seek any statistically significant difference in the levels of alexithymia among the groups included in our sample. Bivariate parametric correlations for continuous variables, using Pearson’s r coefficient, were run on paired samples to establish the nature of any correlations between the alexithymia levels in the young patients and in their mothers. Bivariate parametric correlations for continuous variables, using Pearson’s r coefficient, were also run to explore the relationship between age and alexithymia. Finally, parametric *t* test was applied to find any potential gender difference in alexithymic scores.

Data were analyzed with SPSS software version 19 (Statistical Package for the Social Sciences).

## Results

Concerning alexithymia scores in children, 38 % of the TTH sufferers, 12 % of the M sufferers, and 9 % of the controls were classified as alexithymic (Table 1).

**Table 1:** Distribution of the children’s alexithymia

	Alexithymic n (%)	Borderline n (%)	Non-alexithymic n (%)
M patients (*n* =42)	5 (12 %)	10 (24%)	27 (64 %)
TTH patients (*n* =47)	18 (38 %)	11 (24 %)	18 (38 %)
Controls (*n* =32)	3 (9 %)	13 (41 %)	16 (50 %)

Scores based on the Alexithymia Questionnaire for Children obtained by the M, the TTH and the control groups

*M* migraine, *TTH* tension-type headache

Considering the children’s total score in alexithymia and the subscales separately (F1: difficulty identifying feelings; F2: difficulty describing feelings; F3: externally oriented thinking), significant differences emerged in the total score (F = 3.19; *p* = .04) and in F1 (F = 4.11; *p* = .02), with young TTH patients scoring higher in alexithymia compared to the controls and M patients of the same age (Table 1).

When comparing the mothers’ levels of alexithymia (Table 2), the percentage of mothers classified as alexithymic was 13 % among the mothers of M sufferers, 6 % among the mothers of TTH patients, and 6% among the mothers of controls: differences were not statistically significant (F = 2.92; *p* = .06).

**Table 2:** Distribution of the mothers’ alexithymia

	Alexithymic	Borderline	Non-alexithymic
Mothers of M sufferers (*n* =39)	5 (13 %)	4 (10 %)	30 (77 %)
Mothers of TTH sufferers (*n* =47)	3 (6 %)	4 (9 %)	40 (85 %)
Mothers of controls (*n* =32)	2 (6 %)	9 (28 %)	21 (66 %)

Scores based on the Toronto Alexithymia Scale (TAS-20) obtained by the mothers of young people in the two clinical groups and the control group

*M* migraine, *TTH* tension-type headache

Our second aim was to find a correlation between alexithymic scores of patients (and controls) and alexithymic scores of their mothers. Differently from their children, the mothers of TTH patients and those of the M patients did not score higher in alexithymia compared to the mothers of the controls. However children with high scores appear to be associated with mothers with high scores in the M as well as in the control group: significant correlations between alexithymic total scores of M patients and of their mother and between alexithymic scores of controls and of their mother were found, while the correlation between TTH patients and their mother was not statistically significant (Table 3).

**Table 3:** Mother-child correlations in the alexithymic scores

	Total	F1	F2	F3
	r (p)	r (p)	r (p)	r (p)
M patients and their mothers (*n* = 39)	.44** (<.01)	.39* (.01)	.36* (.02)	.07 (.66)
TTH patients and their mothers (*n* = 47)	.27 (.06)	.29 (.05)*	.09 (.54)	.03 (.83)
Controls and their mothers (*N* = 32)	.38* (.03)	.17 (.34)	.18 (.33)	.24 (.19)

*Alexithymic score*s obtained using the Alexithymia Questionnaire for Children for patients and controls and the Toronto Alexithymia Scale for their mothers, *Total score,* *F1* difficulty identifying feelings, *F2* difficulty describing feelings, *F3* externally oriented thinking

*M* migraine, *TTH* tension-type headache

*p < .05  **p < .01

In terms of gender, there were no statistically significant relations between this variable and the alexithymia scores in our sample, the only exception being the F3 factor in the M group: males suffering from M in fact scored higher in F3 than females (t = 2.71; *p* = 0.01).

In the TTH group only, there was a statistically significant correlation between age and total alexithymia score, F1 and F3 (Table 4).

**Table 4:** Correlations between alexithymia and age

	Total	F1	F2	F3
	r (p)	r (p)	r (p)	r (p)
M patients (*n* = 42)	.12 (.43)	.10 (.52)	−.01 (.93)	.18 (.25)
TTH patients (*n* = 47)	−.43** (<.01)	−.36* (.01)	−.22 (.14)	−.49** (<.01)
Controls (*n* = 32)	−.09 (.63)	.09 (.60)	−.15 (.42)	−.16 (.37)

*Alexithymic* *scores* obtained using the Alexithymia Questionnaire for Children, *Total score,* *F1* difficulty identifying feelings, *F2* difficulty describing feelings, *F3* externally oriented thinking

*M* migraine, *TTH* tension-type headache

*p < .05  **p < .01

## Discussion

Young TTH sufferers scored higher in alexithymia and revealed greater difficulty in identifying feelings (F1) compared to M sufferers and controls of the same age. Previous studies found headache patients to be more alexithymic than controls [19–21]. Concerning adult samples, Wise et al. [20] in comparing 100 adult patients, M or TTH sufferers, with a group of healthy controls, found higher alexithymia scores in the former. However in this study [20] no differences emerged between patients with M and patients with TTH. Yücel et al. [21] demonstrated higher levels of alexithymia in patients aged 18–65 years suffering from episodic or chronic TTH than in healthy controls. No differences in the severity of alexithymia were found in cases experiencing episodic versus chronic TTH. Pini et al. [26], in line with such results found alexithymic characteristics linked to stress symptoms in chronic TTH patients, and to a greater extent in those with medication-overuse headache. There was also preliminary evidence in this sense among preadolescent and adolescent patients with TTH, who scored higher in alexithymic features compared with controls of the same age [19].

Our study confirms a significant relation between TTH and alexithymia but not between M and alexithymia in a child and adolescent sample. A few studies in the adult population have nonetheless revealed a greater extent of alexithymic characteristics in M patients compared to the general population [27–29]. Children and adolescent samples can however yield different results. A possible explanation for the difference between the alexithymia in TTH and in M patients lies in the different pathogenesis of the two disorders. While in M genetic factors may be the most important factors, TTH has a complex multifactorial pathogenesis, which also involves various biological, psychological, social and other factors, especially in childhood and adolescence.

In relation to psychological factors, a recent epidemiologic study [16], reporting data from the WHO’s World Mental Health surveys, demonstrated that a broad range of preexisting mental disorders are likely to increase the risk of subsequent onset of headache in general population samples. A temporal association between preexisting psychiatric and psychological disorders and the onset of headaches may entail different mechanism implied in emotional regulation. While the psychopathology correlated to pediatric headache has been extensively studied [30], the relation between headache and emotional regulation, in particular alexithymia, appears to be much less studied.

Findings on child and adolescent headache patients have disclosed not only a high prevalence of depression, anxiety and behavioural symptoms but even a great extent of somatic concerns, illnesses, stress, and an unhappy atmosphere in the childrearing experience [30–32]. Moreover, preliminary data suggested an association between headache symptoms and insecure attachment [33, 34], which is known to be related to alexithymia and to deficits in affective regulation [35, 36]. Affective regulation is usually acquired in the first childrearing experiences, when children learn how to name and express their affects, sharing them with their primary caregiver, who reflects their emotional expressions in playful interactions [1, 37]. This theoretical premise shapes the hypothesis that the child’s alexithymia may correspond to a similar kind of emotional dysregulation in the mother [19].

The second aim of our study was to explore the mothers’ emotional competence related to that of their offspring, seeking any correlation between the alexithymic scores obtained by the children/adolescents and those obtained by their mothers within our two clinical groups and within the control group. It is worth noting that no statistically significant differences emerged between the three groups of mothers (mothers of M, THT and control groups) as regards alexithymia (overall and considering the subscales F1, F2, F3). Statistically significant correlations were however found between the mothers’ alexithymic scores and those obtained by their children in the M group and in the control group. In the TTH group however the correlation between the mothers’ and children’s alexithymia was significant only for the F1 subscale, difficulty in identifying feelings. The results of our study therefore only partially support the hypothesis advanced by Taylor and Bagby [37] that view alexithymia as an impaired affect regulation acquired in the early years of development within the relationship with the primary caregiver: alexithymia in a child may correspond to a similar kind of emotional dysregulation in the mother. Genetics might also play a part in the etiology of alexithymia, though other social and environmental factors are significant in children’s cognitive-affective development [12–15].

Nevertheless the lack of correlation between TTH patients, the children with the higher level of alexithymia, and their mothers points to a potentially more complex pattern of intergenerational transmission of alexithymia. A child’s alexithymia may not always correspond to a mother’s deficit in emotional competence or may correspond to a kind of maternal emotional dysregulation that differs from alexithymia. This is in line with a previous study conducted on a TTH sample of children and adolescent patients [19] who generally scored higher than their mothers in alexithymia. Moreover the highest alexithymic scores in children seemed to be associated with the lowest scores in their mothers. It is worth noting that even studies concerning different pathologies, such as eating disorders, have found no correspondence between alexithymia in the offspring and in their mothers [38, 39]; on the contrary children’s alexithymia seemed to correspond to mothers’ tendency to overexpress their feelings [38]. These studies suggest that potential deficits in the emotional competence and in affective regulation can be expressed in a different way by the mothers and by their children. The results obtained in this study on TTH patients also point to a nonlinear transmission of alexithymia from mother to child, confirming previous results in children and adolescent samples [19].

In line with this result, the mothers of the three groups (M, TTH and control) showed no differences in alexithymic scores. This means that the mothers of patients with headaches did not show higher levels of alexithymia compared to other mothers. Differences, if any exist, between mothers of TTH and M patients and mothers of the control group, might lie in other aspects of emotional regulation implied in mood and anxiety disorders, which have an high incidence in parents of children with headache [17, 40, 41]. Future studies exploring these aspects may be useful to tackle important stressors and risk factors for the onset of headaches in genetically predisposed children [16, 17].

Exploring any potential relationship between gender and alexithymic traits in young headache sufferers, we found no statistically significant differences between male and females in our sample’s alexithymic traits. The only exception being the F3 factor, where males suffering from M scored higher than females in the M group. Although the small number of males within the samples might have limited the possibility of exploring gender differences in greater depth, these results are in line with literature [23, 42, 43]. Although gender differences were reported by studies conducted on adult samples [44, 45], in adolescent [42, 43] and child [23] samples differences are much less obvious than in an adult population. Concerning the age factor, no differences emerged within the TTH and the control groups, while in the M group the alexithymic score was inversely proportional to age, except for the F2. This is in line with the decay of alexithymia documented from childhood to adolescence [42]. Such an event could be partially explained by the fact that cognitive skills regulating interpersonal relations and affective regulation reach full development in adolescence [19].

The main limitations of this study need to be mentioned in order to pave the way for future research in this field. One of the weaknesses of this study is that the data are based only on self-reported emotional difficulties while, as generally known, a multi-informant evaluation may be more precise and valuable, especially in detecting emotional difficulties, which young people might not always be completely aware of [37]. Nonetheless the Alexithymia Questionnaire for Children’s rewording [23], tailored specifically to children, could in part overcome the limitation due to the difficult self-reported evaluation for younger age groups. Such a self-reported instrument has proven to be useful and handy in conducting this study on two specific child headache samples, where alexithymic characteristics were still largely unexplored. Moreover using two theoretically similar and comparable measures for the child’s (AQC) and the mother’s (TAS-20) alexithymia served to identify a relationship between the two. Subsequent studies deserve to be carried out on the topic to examine in depth the relation between headache symptoms and emotional functioning, using clinical indices and interviewer rated instruments.

A second limitation of the study involves the limited size and the gender distribution of the three samples, where there is a prevalence of females than males. This can in part mirror the fact that there is a marked feminine prevalence of headaches in this age group. Metanalytical data [46] points to a higher risk for the onset of headache symptoms in female compared to male children and adolescents. Moreover this difference might be more marked in preadolescence and in adolescence, in particular for tension-type headache [47]. In this age group the prevalence of daily chronic headaches is even two to three fold higher in girls compared to boys [48–50]. Due to the limited size of the sample, diagnostic distinction among different kinds of M and TTH was taken into account only for descriptive analysis, while inferential statistics would have been less effective given the small number of subjects in the different groups. Comparing specifically chronic TTH patient’s alexithymia to other TTH patients’ could be an interesting objective for a future study conducted on a larger sample of patients.

Another limitation of the study regards the missing data on paternal alexithymia. Future studies should include fathers to further explore the mechanisms involved in family emotional functioning and in the intergenerational transmission of alexithymia [38]. Close family members need to be involved in the assessment of psychological traits and emotional competencies that may constitute a risk or, on the contrary, a protective factor for the onset of the child’s headache [16, 17, 40, 41].

## Conclusions

Given the paucity of publications on the relation between alexithymia and primary headache in children [19] and adolescents [19, 27], our study can significantly contribute by revealing a statistically significant association between alexithymia and TTH. This is consistent with the interpretation of somatic complaints according to a paradigm of deregulated emotion, confirming the conviction that alexithymia creates a condition in which feelings, when not discriminated against, can undergo a process of reinforcement and become a symptom of disease [2–4]. Alexithymia would consequently be a risk factor for the onset of medical or psychiatric, organic or functional disorders [19, 51–53]. Moreover, it would be important to refer to factors that are not strictly organic, such as the psychological dimension, when contextualizing and managing TTH in the child and adolescent age group. These considerations converge towards a holistic and multidisciplinary approach to tackle the problem of headache.

Literature offers no data on the prevalence of alexithymic traits in children who suffer from M as opposed to TTH. The present study indicates that young M sufferers tend to be less alexithymic than TTH child and adolescent patients. Despite the obvious limitation of having a small sample, this study has generated some novel findings that may be a preliminary step and a starting point for further investigations in this field, oriented towards a comprehensive multidisciplinary approach in the care of children and adolescents suffering from headaches.

## Abbreviations

TTH: Tension-type headache
M: Migraine
ICHD: International Classification of Headache Disorders
TAS-20: Toronto Alexithymia Scale
F1: Difficulty identifying feelings
F2: Difficulty describing feelings
F3: Externally oriented thinking
